# LC-ESI-MS/MS Identification of Biologically Active Phenolic Compounds in Mistletoe Berry Extracts from Different Host Trees

**DOI:** 10.3390/molecules22040624

**Published:** 2017-04-12

**Authors:** Wioleta Pietrzak, Renata Nowak, Urszula Gawlik-Dziki, Marta Kinga Lemieszek, Wojciech Rzeski

**Affiliations:** 1 Department of Pharmaceutical Botany, Medical University of Lublin, Chodźki 1 Str., 20-093 Lublin, Poland; wioleta.pietrzak@umlub.pl; 2 Department of Biochemistry and Food Chemistry, University of Life Sciences, Skromna 8 Str., 20-704 Lublin, Poland; urszula.gawlik@up.lublin.pl; 3 Department of Medical Biology, Institute of Rural Health in Lublin, Jaczewskiego 2, 20-090 Lublin, Poland; martalemieszek@gmail.com; 4 Department of Virology and Immunology, Maria Curie Skłodowska University, Akademicka 19, 20-033 Lublin, Poland; wojciech.rzeski@poczta.umcs.lublin.pl

**Keywords:** antioxidant activity, antiproliferative activity, cytotoxicity, LC-MS/MS (mass spectrometry), phenolic compound, *Viscum album* L.

## Abstract

A new, rapid, sensitive and selective liquid chromatography-electrospray ionization-tandem mass spectrometry (LC-ESI-MS/MS) method was developed to determine the content of flavonoid aglycones and phenolic acids in mistletoe berries (*Viscum album* L.) harvested from six different Polish host trees. Additionally, the total phenolic content (TPC) and total flavonoid content (TFC) as well as an antioxidant and antiproliferative activity were evaluated for the first time. The plant material was selectively extracted using ultrasound assisted maceration with methanol/water (8:2) solution. The obtained TPC and TFC results varied from 7.146 to 9.345 mg GA g^−1^ and from 1.888 to 2.888 mg Q g^−1^ of dry extracts, respectively. The LC-ESI-MS/MS analysis demonstrated the highest content of phenolic acids in mistletoe berries from *Populus nigra* ‘Italica’ L. and flavonoid aglycones in mistletoe berries from *Tilia cordata* Mill. (354.45 µg and 5.955 µg per g dry extract, respectively). The moderate antioxidant activity of investigated extracts was obtained. The studies revealed that the examined extracts decreased the proliferation of human colon adenocarcinoma cells line LS180 in a dose-dependent manner without cytotoxicity in the human colon epithelial cell line CCD 841 CoTr. Moreover, the obtained results suggest considerable impact of polyphenols on the anticancer activity of these extracts.

## 1. Introduction

Mistletoe (*Viscum album*) is a perennial evergreen shrub that grows as a hemiparasite on woody plant species. The plant contains large amounts of active components such as lectins, viscotoxins, phenylpropanes, lignans, flavonoids, amines and polysaccharides. Literature reports have demonstrated its antioxidant, anticancer, anti-inflammatory, and antibacterial properties as well as antidiabetic, antiepileptic, immunostimulatory and antiviral activity [1,2,3,4,5,6]. Mistletoe has been evaluated as a treatment for people with numerous types of cancers in several clinical studies [7,8,9], and the findings have suggested that adjuvant treatment of cancer patients with mistletoe extracts is associated with a better survival rate, alleviated conventional therapy side effects and improved quality of life. Among the different types of cancer, human colon cancer is the third most commonly diagnosed malignant disease [10]. Over the last decade there has been a great deal of evidence from genetic, pharmacological, and epidemiological data supporting the notion of a connection between antioxidant, and anti-inflammatory activity and tumorigenesis. High concentrations of free radicals are an important risk factor for the development of colon cancer. Anti-oxidant activity affects every facet of tumor development and might influence the efficacy of cancer therapies [11].

A variety of bioactive compounds, especially phenolic compounds, have been reported to possess important biological properties such as anticancer, antiviral, antioxidant and anti-inflammatory activities [12,13]. Some natural products are now being used for cancer prevention and/or therapy, and as adjuvants for conventional therapies. 

The literature reports the beneficial effects of supportive care mistletoe extract therapy within adjuvant therapy (AT) protocols and long-term treatment with mistletoe extracts in stage I-III colorectal carcinoma patients, particularly improvement in AT-ADRs and symptoms and possible extension of disease-free survival [14]. The above information and sparse data regarding mistletoe berries in the literature [15,16,17,18] suggest that research into this subject area could provide valuable data about the chemical composition of mistletoe berries and their use.

The aim of the present work was to determine the chemical composition of mistletoe berries from different host trees using liquid chromatography-electrospray ionization-tandem mass spectrometry (LC-ESI-MS/MS). For the first time phenolic acids and flavonoid aglycones were analyzed in the extracts of mistletoe berries; the latter were assayed with a newly developed method. Moreover, the total phenolic and flavonoid contents of *Viscum* L. extracts were measured. The antioxidant activity of extracts was measured by four methods: reducing power (RED), metal chelating power (CHEL), inhibition of linoleic acid peroxidation (LPO) and free radical scavenging ability (ABTS^•^).

Additionally, the antiproliferative and cytotoxic activities of the extracts were determined in human colon cancer cells LS180 and human colon epithelial cells CCD 841 CoTr, respectively. The antiproliferative activity of extracts was tested using MTT and BrdU assays while cytotoxicity was verified using the LDH test. Such trials are valuable to determine whether ethanolic extracts from mistletoe berries (without toxic proteins and lectins) could be useful in the treatment of some kinds of diseases, especially cancers.

## 2. Results and Discussion

The purpose of the present study was to determine the phenolic acid and flavonoid aglycone contents and antioxidant activity of extracts from mistletoe berries harvested from different host trees. Phenolic compounds are widely distributed in the plant kingdom and are the most abundant secondary metabolites found in plants. The biological activity of phenolics is related, at least in part, to their antioxidant properties, due to both their free-radical scavenging activity and metal chelating properties [19,20,21]. There is growing scientific interest in the biological properties of polyphenols and flavonoids beneficial for prevention of age-related diseases, including cardiovascular diseases and cancer [22,23,24]. As previously mentioned, there are only a few scientific studies regarding mistletoe berries. Therefore, our findings aim to yield new valuable data on the chemical composition of *Viscum album* L.

The total phenolic and flavonoid contents of mistletoe extracts are shown in Table 1. The total phenolic content varied from 4.78 to 10.43 mg GA g^−1^ of dry extract and the total flavonoid content–from 0.27 to 0.428 mg Q g^−1^ of dry extract. The results of the present study demonstrated the highest amount of polyphenols and favonoids in extracts of mistletoe berries collected from *Populus nigra* “Italica” L. and *Sorbus aucuparia* L.

Liquid chromatography coupled with mass spectrometry (LC-MS) is one of the most effective analytical tools for organic compound analysis and has many advantages. It provides easy identification of eluted analytes as well as structural and molecular weight information, reduces sample preparation time; quantitative and qualitative data can be obtained easily from limited optimization of distributions [24,25,26].

**Figure 1:**
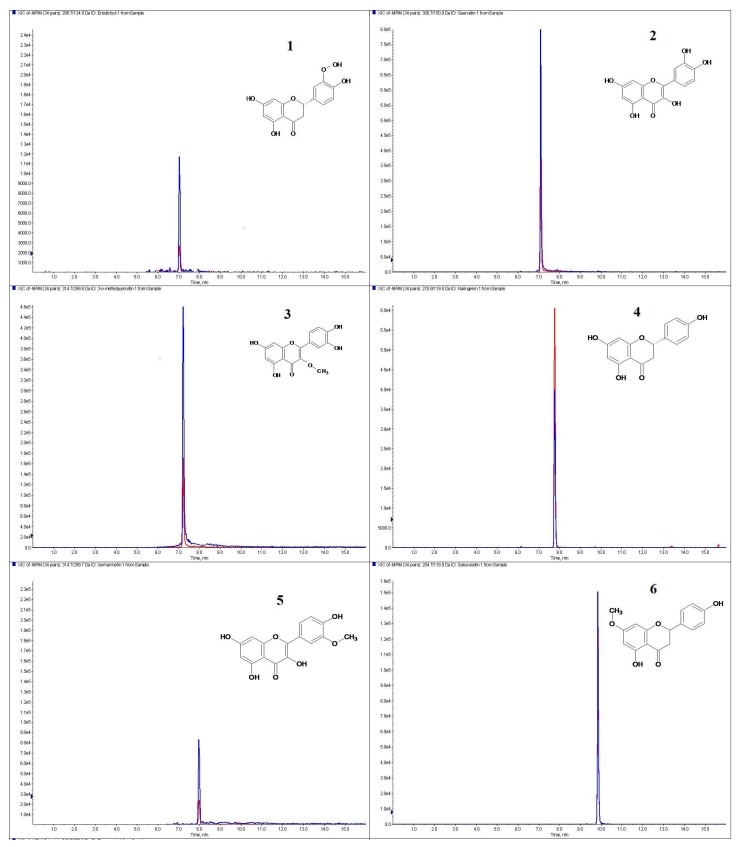

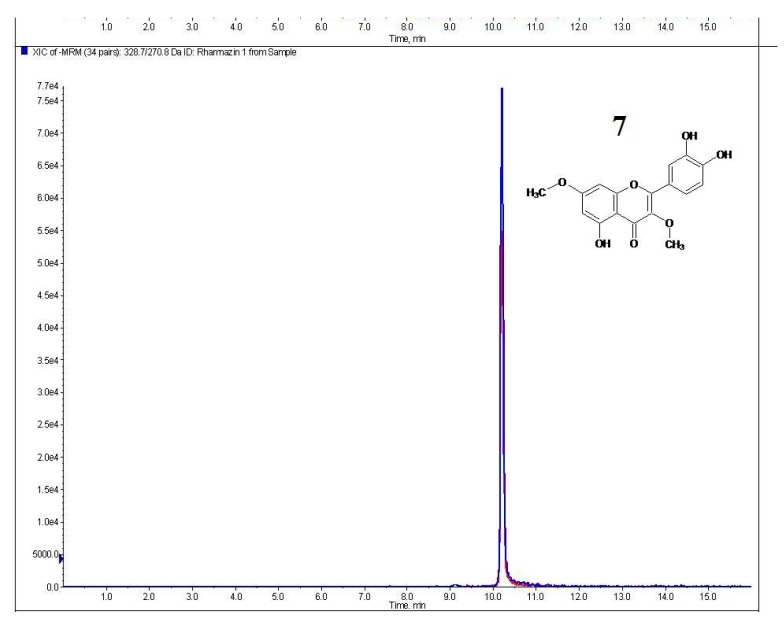
The chromatograms in MRM mode of standards solution of flavonoid aglycones occurring in mistletoe berries: **1**–Eriodictiol; **2**–Quercetin; **3**–3-o-Methylquercetin; **4**–Naringenin; **5**–Isorhamnetin; **6**–Sakuranetin; **7**–Rhamnazin.

A new method for the determination of flavonoid aglycones by LC-ESI-MS/MS was developed and applied for the first time. Chromatograms in MRM mode of standard solutions of flavonoid aglycones occurring in mistletoe berries are shown in Figure 1.

The majority of the tested compound were obtained from *Tilia cordata* Mill. and *Populus nigra* ‘Italica’ L. mistletoe berries (5.955 and 2.161 µg per g dry extract respectively) (Table 2).

Among free flavonoid aglycones, large amounts of naringenin (2.319 ± 0.046 µg per g dry extract), isorhamnetin (1.794 ± 0.09 µg per g dry extract), rhamnazin (1.223 ± 0.061 µg per g dry extract) and eriodictiol (0.201 ± 0.01 µg per g dry extract), were found in mistletoe berries from *Tilia cordata* Mill. A flavonoid aglycone characteristic of mistletoe berries from *Populus nigra* ‘Italica’ L. was sakuranetin (0.109 ± 0.0003 µg per g dry extract). These extracts also contained significant amounts of naringenin (0.427 ± 0.0001 µg per g dry extract), 3-*O*-methylquercetin (0.532 ± 0.004 µg per g dry extract), rhamnazin (0.472 ± 0.024 µg per g dry extract) and quercetin (0.056 ± 0.003 µg per g dry extract). Most of phenolic acids obtained by the LC-ESI-MS/MS method (Table 3) were determined for mistletoe berries from *Sorbus aucuparia* L. and *Populus nigra* ‘Italica’ L. (respectively 351.13 and 354.45 µg per g dry extract); the lowest amount was found in mistletoe berries from *Pinus sylvestris* L. (*Viscum album* subsp. *austriacum*).

The berries harvested from *Fraxinus pensylvanica* Marsh. contained large amounts of gallic acid (23.17 ± 0.58 µg per g dry extract), protocatechuic acid (80.79 ± 0.57 µg per g dry extract) and syringic acid (98.6 ± 0.69 µg per g dry extract). In other extracts, gallic acid was only present in trace amounts and the protocatechuic acid content ranged from trace amounts to a maximum value of 49.64 ± 0.50 µg per g dry extract for mistletoe berries from *Malus domestica* Borkh.

A characteristic compound for mistletoe berries from *Malus domestica* Borkh. and *Pinus sylvestris* L. (*Viscum album* subsp. *austriacum*) was ferulic acid (13.64 ± 0.67 and 11.81 ± 0.01 µg per g dry extract respectively). The extract of mistletoe from *Populus nigra* ‘Italica’ L. was characterized by the presence gentisic acid (3.26 ± 0.16 µg per g dry extract) and large amounts of sinapic and syringic acid (112.17 ± 1.79 and 137.99 ± 1.24 µg per g dry extract respectively).

The extracts from mistletoe berries coming from *Sorbus aucuparia* L. contained large amounts of caffeic acid (34.74 ± 0.52 µg per g dry extract), vanillic acid (43.9 ± 1.36 µg per g dry extract), sinapinic acid (107.08 ± 1.07 µg per g dry extract) and syringic acid (121.54 ± 1.58 µg per g dry extract).

The results of tests obtained for mistletoe berries from *Tilia cordata* Mill were noteworthy. In this case, synapinic acid was the characteristic phenolic acid present in large quantities (84.80 ± 1.36 µg per g dry extract).Moreover, biological research of mistletoe berries was carried out. The antioxidant activity of extracts was measured by four methods: reducing power (RED), metal chelating power (CHEL), inhibition of linoleic acid peroxidation (LPO) and free radical scavenging ability (ABTS^•^)–Table 4.

The extract of mistletoe berries harvested from *Tilia cordata* Mill. showed the highest antioxidant activity measured by RED, LPO and CHEL methods. The highest antioxidant activity tested using ABTS^•^ was determined for the extract of mistletoe berries harvested from *Sorbus aucuparia* L. *Fraxinus pensylvanica* Marsh. and *Populus nigra* ‘Italica’ L. (EC_50_ 7.72, 8.06 and 8.17 mg mL^−1^ of extract respectively).

The results of statistical analysis suggested that the phenolic and flavonoid content correlated with the results of antioxidant activity measured by the ABTS method (extracts which contained high amounts of phenolic and flavonoid compounds showed a low EC_50_ value, hence higher antioxidant activity—Table 5). Statistical analysis suggests that the antiradical activity is in 49% determined by the total phenolic content, in 65% by total flavonoid content and in 95% by total phenolic acids content (R^2^ × 100%).

**Figure 2:**
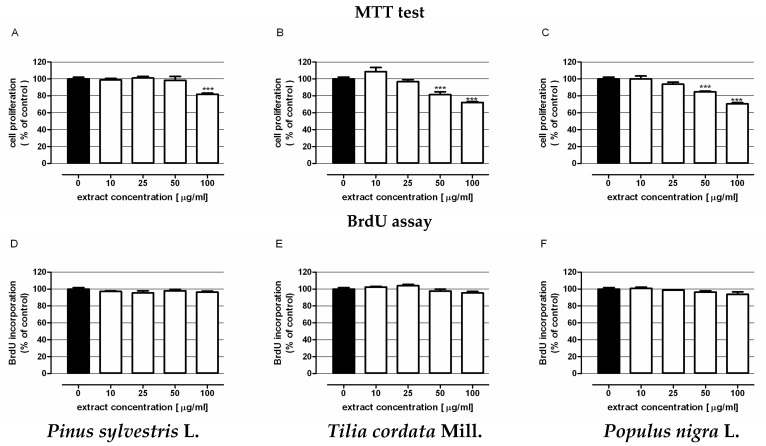
The influence of mistletoe berries extracts on the proliferation of human colon adenocarcinoma cell line LS180. Cells were exposed for 96 h (**upper panel**) or 48 h (**bottom panel**) to extracts isolated from berries of mistletoe harvested from *Pinus sylvestris* L. (**A**,**D**); **Tilia cordata** Mill. (**B**,**E**); *Populus nigra* L. ‘Italica’ (**C**,**F**). Cell proliferation was quantified by MTT test (upper panel) or BrdU assay (**bottom panel**). Results are presented as the mean ± SEM of 4–5 measurements. *** *p* < 0.001 vs. control, one-way ANOVA test; post test: Tukey.

The effects of extracts on human colon cancer cell proliferation were examined by MTT and BrdU assays, performed after 96 or 48 h, respectively, of LS180 cell treatment with the assessed compounds. The MTT assay results (upper panel of Figure 2) revealed the antiproliferative abilities of the studied extracts. The extracts isolated from mistletoe berries harvested from linden (Figure 2B) and poplar (Figure 2C) suppressed colon cancer cell proliferation in a dose-dependent manner, starting with the concentration of 50 μg/mL. At the highest concentration tested (100 μg/mL), a decrease in cell proliferation of approximately 30% versus control conditions was observed. The extracts obtained from mistletoe growing on *Pinus sylvestris* L. (Figure 2A) inhibited cancer cell proliferation only when the highest investigated concentration was used. The IC_50_ values calculated for all extracts were 1 mg/mL, 164 µg/mL and 202 µg/mL, respectively.

The antiproliferative properties of extracts were further verified by the more sensitive and specific BrdU assay, which enables one to determine the influence on DNA synthesis in proliferating cells. As presented in the bottom panel of Figure 2, 48 h of cancer cells exposure to the extracts did not affect DNA synthesis.

In vitro studies have revealed that ethanol extracts isolated from mistletoe berries decreased the proliferation of colon cancer cells in a dose-dependent manner. The effect was observed after 96 h of cells exposure and was attributed to altered mitochondrial activity. The changes were dependent on the tree species from which mistletoe was collected and used for extract preparation. The best results were obtained from the material harvested from *Tilia cordata* Mill. and *Populus nigra* ‘Italica’ L. Otherwise, the effects of extracts on the DNA synthesis in colon cancer cells were not observed.

**Figure 3:**
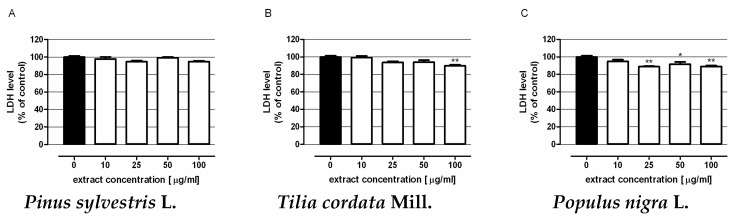
Cytotoxicity of mistletoe berries extracts in human colon epithelial cell line CCD 841 CoTr. Cells were exposed for 48 h to extracts isolated from berries of mistletoe harvested from *Pinus sylvestris* L. (**A**); **Tilia cordata** Mill. (**B**); *Populus nigra* L. ‘Italica’ (**C**). Extract cytotoxicity was measured by means of the LDH assay. Results are presented as the mean ± SEM of 4 measurements. * *p* < 0.05 vs. control, ** *p* < 0.01 vs. control, one-way ANOVA test; post test: Tukey.

Extract cytotoxicity was examined in human colon epithelial cells (CCD 841 CoTr) by means of an LDH test. Cells were exposed for 48 h to culture medium alone (control) or the extracts in concentrations ranging from 10 to 100 µg/mL. All tested extracts were nontoxic to colon epithelial cells in the whole range of investigated concentrations (Figure 3). Furthermore extracts isolated from mistletoe berries harvested from *Tilia cordata* Mill. in the highest concentration (Figure 3B) and *Populus nigra* ‘Italica’ L. in the concentration range 25–100 µg/mL (Figure 3C) significantly reduced the level of LDH release from treated cells.

Since anticancer agents should not be toxic for normal cells, the cytotoxic effects of extracts were evaluated in human intestinal epithelial cells. The findings revealed that the substances tested did not affect the integrity of colon epithelial cell membranes. Furthermore, the extracts isolated from mistletoe growing on *Tilia cordata* Mill. and *Populus nigra* ‘Italica’ L. were found to be able to reduce the level of LDH release from cells, which might indicate protective properties of the substances in question.

It has been reported that phenolic compounds possess important biological properties such as anticancer, antiviral, antioxidant and anti-inflammatory activities [13,27]. A substantial body of evidence supports the theory of anticancer properties of phenolic acids, and although the mechanisms are still not fully understood, they may include scavenging of free radicals, induction of enzymes involved in the metabolism of xenobiotics, regulation of gene expression, modulation of cellular signaling pathways including those involved in DNA damage repair, cell proliferation, apoptosis and invasion [12,28].

The large amounts of naringenin, isorhamnetin and rhamnazin were found in extracts from mistletoe berries from *Tilia cordata* Mill. and *Populus nigra* ‘Italica’ L. Moreover, in the extract from mistletoe berries from *Populus nigra* ‘Italica’ L. the largest content of phenolic acids were determined. In vitro studies showed that ethanolic extracts from mistletoe berries from *Tilia cordata* Mill. and *Populus nigra* ‘Italica’ L. decreased the proliferation of colon cancer cells to the greatest extent.

Many studies have shown various pharmacological effects including antioxidant, anti-inflammatory, antimutagenic and anticancer activity of naringenin (NAR) [29,30,31,32]. There is growing evidence that naringenin induces apoptosis in human colon cancer cells, showing no toxic effects on normal cells at a similar dose.

Activating transcription factor 3 (ATF3) is associated with apoptosis in human colon cancer. Song et al. [33] demonstrated that naringenin increases the transcriptional activation and increase of ATF3 protein level through p38 activation, and NAR-mediated ATF3 expression contributes, at least in part, to apoptosis in human colon cancer cells.

Moreover, Song et al. [34] demonstrated that NAR-induced proteasomal degradation of cyclin D1 might inhibit the cell proliferation in human colorectal cancer cells. Their study provides information on the apoptotic effect and the potential molecular mechanism of naringenin.

Likewise Leonardi et al. [35] indicated that naringnenin was promising as a naturally occuring chemopreventive agents against colon carciogenesis.

The beneficial effects of isorhamnetin, such as anti-inflammatory, antioxidant and anti-cancer have been reported in various tissues [36,37,38]. Seo et al. [39] determined that the antioxidant activity of isorhamnetin was correlated with its anti-cancer effects on colorectal cancer cells. The results demonstrated that isorhamnetin inhibits reactive oxygen species (ROS)-mediated hypoxia-induced hypoxia inducible factor-1α (HIF-1α) accumulation, which contributes to its anti-metastatic efficacy.

Furthermore, it has been found that isorhamnetin (IH) exerted a strong antiproliferative effect and increased cytotoxicity in combination with chemotherapeutic drugs. IH also inhibited the migratory/invasive properties of gastric cancer cells [38,40]. Previous studies and the preclinical evaluations suggest the antiproliferative and apoptogenic effects of rhamnazin [41,42].

To sum up, the literature findings suggest that naringenin, isorhamnetin and rhamnazin could be used as anti-cancer agents, particularly in colon cancer. Moreover, the presented mechanisms can explain the anti-cancer efficacy of various natural compounds with antioxidant effects.

As previously mentioned mistletoe has been evaluated as a treatment for people with numerous kinds of cancers including colon cancer [9]. The literature reports of the beneficial effect of supportive care mistletoe extract Iscador (ISC) therapy within adjuvant therapy (AT) protocols and long-term ISC treatment in stage I-III colorectal carcinoma patients, particularly improvement in AT-ADRs and symptoms and possible extension of disease-free survival [14].

It is worth mentioning that our studies relate to ethanolic extracts from mistletoe berries, without toxic proteins and lectins that are present in aqueous extract of mistletoe. Finally it should also be emphasized that the available literature lacks studies regarding contents of phenolic acids and flavonoid aglycones or antioxidant and biological activities of mistletoe berries.

## 3. Materials and Methods

### 3.1. Plant Material

Berries of *V. album* subsp. *album* were harvested from different host trees, i.e., *Populus nigra* L. ‘Italica’, *Fraxinus pennsylvanica* Marsh., *Malus domestica* Borkh., *Sorbus aucuparia* L., *Tilia cordata* Mill. and *Pinus sylvestris* L. growing close to the Lublin region (Poland) during the winter of 2013/14. The plant material was air-dried at room temperature and powdered.

### 3.2. Extraction Method

Portions of shredded mistletoe berries (10 g) were extracted using ultrasound assisted maceration at room temperature with 8:2 methanol/water solution (50 mL). The plant material was extracted thrice, 15 min each time. In all cases, the extracts were filtered, evaporated to dryness under vacuum and lyophilized in a Free Zone 1 apparatus (Labconco, Kansas City, KS, USA). The residue was weighted and placed in refrigerator (−30 °C). Immediately before analysis the extracts were redissolved in the same solvent as the one used for extraction to obtain a stock solutions. All samples were prepared in triplicate. Before LC-MS analysis, each test solution was filtered through a 0.45 μm membrane filter.

### 3.3. Total Phenolic and Flavonoid Content

The total phenolic content (TPC) was determined according to a modified Folin-Ciocalteu method described earlier [43]. The absorbance was measured at 680 nm after a 20-min incubation. Determinations were carried out using a standard curve prepared for gallic acid and the results were expressed as mg of gallic acid per 1 g of dry extract (GAE–gallic acid equivalent). The total flavonoid content (TFC) was determined according to the method described by Lamaison and Carret [44] with some modifications [45]. The absorbance was read at 430 nm after a 30-min incubation against a blank solution containing methanol instead of the test sample. The results were expressed as mg quercetin (Q) per 1 g of dry extract. All measurements were performed using an Infinite Pro 200F microplate reader (Tecan Group Ltd., Männedorf, Switzerland).

### 3.4. LC-ESI-MS/MS Analysis

#### 3.4.1. Chromatographic Conditions and Apparatus

Phenolic acids and flavonoid aglycones were determined by reversed-phase high-performance liquid chromatography and electrospray ionization mass spectrometry (LC-ESI-MS/MS). An Agilent 1200 Series HPLC system (Agilent Technologies, Santa Clara, CA, USA) equipped with a binary gradient solvent pump, a degasser, an autosampler and a column oven connected to a 3200 QTRAP Mass spectrometer (AB Sciex, Framingham, MA, USA) was used.

The contents of free flavonoid aglycones were determined by a newly developed, simple and rapid method using liquid chromatography-electrospray ionization-tandem mass spectrometry. The compounds were separated at 25 °C on a Zorbax SB-C18 column (2.1 × 50 mm, 1.8-μm particle size; Agilent Technologies). The injection volume was 5 μL. Gradient elution was applied using water containing 0.1% HCOOH (A) and acetonitrile (B). The flow rate was 200 μL/min and the gradient was as follows: 0–0.5 min 5% B; 1–4 min 40% B; 5–9 min 50% B; 11–12 min 70% B; 12.5–13.5 min 90% B; 15–19 min 5% B. Contents of phenolic acids were determined by the LC-ESI-MS/MS method previously described by Nowacka et al. [46].

#### 3.4.2. Optimisation of Quantitative Analysis Parameters

The 3200 QTRAP MS/MS system and an electrospray ion source in the negative mode were used. The optimal mass spectrometer parameters were determined experimentally and for analysis flavonoid aglycones were as follows: curtain gas 20 psi, capillary temperature 550 °C, nebulizer gas 30 psi, negative ionization mode source voltage-4500 V. Nitrogen was used as a curtain and collision gas. The data were acquired and processed using Analyst 1.5 software from AB Sciex (Framingham, MA, USA).

Multiple reaction monitoring (MRM) was used for quantitative analysis of compounds. The analytes were identified by comparing the retention times and m/z values obtained by MS and MS2 with the mass spectra of the corresponding standards tested under the same conditions. The calibration curves obtained in the MRM mode were used for quantification of all analytes. The identified flavonoid aglycones were quantified based on their peak areas and comparison with a calibration curve for the corresponding standards. Linearity ranges for calibration curves were specified.

The limits of detection (LOD) and quantification (LOQ) for phenolic acids and flavonoids aglycone were determined at a signal-to-noise ratio of 3:1 and 10:1, respectively, by injecting a series of dilute solutions of known concentrations. Table 6 and Table 7 summarize the optimized parameters for quantification of flavonoid aglycones.

### 3.5. Biological Activity

#### 3.5.1. Antioxidant Activity 

The antioxidant activity of extracts was measured by four methods. The antiradical activity was assayed using an improved ABTS^•^ decolorization assay [47,48]. The absorbance was measured at 734 nm. The extract ability to quench the ABTS^•^ free radical was determined using the following equation:

Scavenging% = ((AC − AA)/AA) × 100(1)

where AC is the absorbance of control and AA is the absorbance of sample.

Reducing power (RED) was determined by the method of Oyaizu [49] The absorbance was measured at 700 nm with a spectrophotometer (Lambda 40, Perkin-Elmer, Waltham, MA, USA). Increased absorbance of the reaction mixture indicated increased reducing power.

Metal chelating power (CHEL) was determined by the method of Guo et al. [50]. Subsequently, the absorbance of the solution was measured spectrophotometrically at 562 nm. The percentage of inhibition of ferrozine-Fe^2+^ complex formation was calculated according to the following formula:

Inhibition% = (1 − (Ap/Ac)) × 100(2)

where Ac is the-absorbance of control and Ap–the absorbance in the presence of sample.

The degree of inhibition of the hemoglobin-catalyzed linoleic acid peroxidation (LPO) was determined according to Kuo and Sun [51]. The absorbance of sample (As) was measured at 480 nm with a spectrophotometer for 5 min. The absorbance of blank (A0) was determined without hemoglobin added to the reaction mixture; the absorbance of control (A100) was measured with no sample added to the above mixture. Thus, the antioxidative activity of the sample was calculated as:

AA% = (1 − (As − A0)/(A100 − A0)) × 100(3)

All the antioxidant activity results were expressed as EC_50_ values, the extract concentration providing 50% of the activity in a dose-dependent manner.

#### 3.5.2. Examination of Extract Antiproliferative Activity

The human colon adenocarcinoma cell line LS180 was obtained from the European Collection of Cell Cultures (ECACC, Centre for Applied Microbiology and Research, Salisbury, UK). LS180 cells were grown in a 1:1 mixture of DMEM (Sigma-Aldrich, St. Louis, MO, USA) and Ham’s F-12 nutrient mixture (Sigma-Aldrich) supplemented with 10% foetal bovine serum (FBS) (Sigma-Aldrich), penicilin (100 U/mL) (Sigma-Aldrich) and streptomycin (100 μg/mL) (Sigma-Aldrich). Cells were maintained in a humidified atmosphere of 95% air and 5% CO_2_ at 37 °C.

The effects of extracts on cancer cells proliferation were determined by the MTT metabolism assay and BrdU incorporation immunoassay. The BrdU test was conducted using Cell Proliferation ELISA BrdU according to the manufacturer’s instructions (Roche Diagnostics GmbH, Penzberg, Germany). Both assays were performed in human adenocarcinoma cells (LS180). Details of the procedures are described elsewhere [52].

#### 3.5.3. Assessment of Cytotoxicity of Extracts

The human colon epithelial cell line CCD 841 CoTr was purchased from American Type Culture Collection (ATCC, Manassas, VA, USA). CCD 841 CoTr cells were grown in DMEM (Sigma-Aldrich) supplemented with 10% foetal bovine serum (FBS) (Sigma-Aldrich), penicillin (100 U/mL) (Sigma-Aldrich) and streptomycin (100 μg/mL) (Sigma-Aldrich). Cells were maintained in a humidified atmosphere of 95% air and 5% CO_2_ at 33 °C. The cytotoxicity of extracts was evaluated using the colorimetric test based on the measurement of lactate dehydrogenase (LDH) release. Determinations were performed in human colon epithelial cells (CCD 841 CoTr) using a LDH-based in vitro toxicology assay kit (Sigma-Aldrich). Details of the procedure were described elsewhere [52].

### 3.6. Statistical Analysis

All results were expressed as a mean ± standard error of the mean (SEM) of three replications for each extract tested. Moreover the Pearson correlation coefficient and R^2^ coefficient were applied to show relationship between total phenolic content (TPC), total flavonoid content (TFC) total phenolic acids content (LC-ESI-MS/MS), total flavonoid aglycones (LC-ESI-MS/MS) and EC_50_ (ABTS^•^) for mistletoes berries extracts from different host trees.

Calculations were performed in STATISTICA 10.0 (StatSoft PolandCracow, Poland). Statistical analysis was performed using the one-way ANOVA with the Tukey post-hoc test and column statistics used for comparisons. Significance was accepted at *p* < 0.05. The IC_50_ value of biological activity (concentration causing proliferation inhibition by 50% compared to control) was calculated according to the Litchfield-Wilcoxon method [53].

## 4. Conclusions

The chemical composition and biological activity of mistletoe berries (*Viscum album* L.) collected from different host plants were studied for the first time. Our findings demonstrated that the extracts of *V. album* contained large amounts of polyphenolic compounds. Phenolic acids and flavonoids may be the characteristic compounds indicating a subspecies of the host tree from which the mistletoe was collected. In vitro studies have revealed that ethanol extracts isolated from mistletoe berries decreased the proliferation of colon cancer cells in dose-dependent manner; the best results were obtained from *Tilia cordata* Mill. and *Populus nigra* ‘Italica’ L materials. Moreover, the substances assessed did not affect the integrity of colon epithelial cell membranes and extracts isolated from mistletoe growing on *Tilia cordata* Mill. and *Populus nigra* ‘Italica’ L. were able to reduce the level of LDH release from cells, which might prove the protective properties of the substances tested.

Moreover, this work presented newly developed method of phenolic compound using simple, rapid, and reliable liquid chromatography-electrospray ionization-tandem mass spectrometry. Tge present studies suggest that the phenolic acids and flavonoids might have potential as chemopreventive agents against colon tumor development.

## Figures and Tables

**Table 1:** Total phenolic content (TPC) and flavonoid content (TFC) of methanol extracts from mistletoe berries harvested from various host trees.

Species	Host Tree	TPC (mg GA g^−1^ of dry extract)	TFC (mg Q g^−1^ of dry extract)
*V.* *album*	*Fraxinus pensylvanica* Marsh.	8.82 ± 0.18 ^ns^	0.365 ± 0.005 ^ns^
	*Malus* *domestica* Borkh.	6.71 ± 0.32 *	0.356 ± 0.006 ^ns^
	*Sorbus* aucuparia L	9.66 ± 0.28 ^ns^	0.428 ± 0.007 ^ns^
	*Populus* *nigra* ‘Italica’ L.	10.43 ± 0.20	0.404 ± 0.017
	*Tilia* *cordata* Mill.	4.78 ± 0.09 *	0.286 ± 0.007 ^ns^
*V. austriacum*	*Pinus sylvestris* L.	7.11 ± 0.20 ^ns^	0.270 ± 0.007 ^ns^

Values are presented in mean ± SEM, *n* = 3 and evaluated by one-way ANOVA (post test: Tukey) to compare each extract to *Populus nigra* ‘Italica’ L. extract. Differences were considered to be statistically significant if *p* < 0.05; ns–no significant differences; * *p* < 0.05.

**Table 2:** Flavonoid aglycones contents in extracts from mistletoe berries collected from different trees host. Values are presented in mean ± SEM, *n* = 3.

Compound	*V. album*					*V. austriacum*
	*Fraxinus* *pensylvanica* Marsh.	*Malus domestica* Borkh.	*Sorbus aucuparia* L.	*Populus nigra* ‘Italica’ L.	*Tilia cordata* Mill.	*Pinus sylvestris* L.
Flavonoid aglycones (µg per g dry extracts)						
Taxifolin	trace ^a^	- ^b^	trace ^a^	trace ^a^	trace ^a^	- ^b^
Myricetin	- ^b^	- ^b^	- ^b^	- ^b^	- ^b^	- ^b^
Morin	- ^b^	- ^b^	- ^b^	- ^b^	- ^b^	- ^b^
Eriodictiol	0.011 ± 0.001	trace ^a^	0.011 ± 0.001	0.014 ± 0.001	0.201 ± 0.01	0.001 ± 0.0001
Luteolin	- ^b^	- ^b^	- ^b^	trace ^a^	- ^b^	- ^b^
Quercetin	trace ^a^	0.025 ± 0.0001	0.006 ± 0.0001	0.056 ± 0.003	trace ^a^	0.026 ±0.0001
3-*O*-Methylquercetin	0.255 ± 0.002	0.163 ± 0.008	0.510 ± 0.020	0.532 ± 0.004	0.409 ± 0.02	0.009 ± 0.0001
Apigenin	- ^b^	- ^b^	trace ^a^	trace ^a^	trace ^a^	trace ^a^
Naringenin	0.111 ± 0.002	0.102 ± 0.002	0.427 ± 0.0001	0.681 ± 0.014	2.319 ± 0.046	0.251 ± 0.013
Kaempferol	- ^b^	- ^b^	- ^b^	trace ^a^	- ^b^	trace ^a^
Isorhamnetin	trace ^a^	0.099 ± 0.005	0.106 ± 0.005	0.297 ± 0.006	1.794 ± 0.09	0.013 ± 0.001
Rhamnetin	trace ^a^	trace ^a^	trace ^a^	trace ^a^	trace ^a^	trace ^a^
Chrysin	- ^b^	- ^b^	- ^b^	- ^b^	- ^b^	- ^b^
Sakuranetin	0.014 ± 0.0001	0.001 ± 0.0002	0.054 ± 0.003	0.109 ± 0.0003	0.009 ± 0.0001	0.004 ± 0.0001
Prunetin	- ^b^	- ^b^	- ^b^	- ^b^	- ^b^	- ^b^
Rhamnazin	0.009 ± 0.0001	0.067 ± 0.003	0.122 ± 0.002	0.472 ± 0.024	1.223 ± 0.061	0.008 ± 0.0001
TOTAL	0.4	0.457	1.236	2.161	5.955	0.312

^a^ trace–trace amounts; - ^b^ not detected.

**Table 3:** Phenolic acid contents in extracts from mistletoe berries collected from different trees host. Values are presented in mean ± SEM, *n* = 3.

Compound	*V. album*					*V. austriacum*
	*Fraxinus* *pensylvanica* Marsh.	*Malus domestica* Borkh.	*Sorbus aucuparia* L.	*Populus nigra* ‘Italica’ L.	*Tilia cordata* Mill.	*Pinus sylvestris* L.
Phenolic Acids (µg per g dry Extracts)						
Gallic acid	23.17 ± 0.58	trace ^a^	trace ^a^	trace ^a^	trace ^a^	- ^b^
Protocatechuic acid	80.78 ± 0.57	40.88 ± 1.59	29.03 ± 1.45	31.07 ± 1.55	trace ^a^	trace ^a^
Gentisic acid	- ^b^	- ^b^	trace ^a^	3.26 ± 0.16	trace ^a^	trace ^a^
4-OH-benzoic acid	11.23 ± 0.11	5.20 ± 0.14	2.72 ± 0.14	0.79 ± 0.04	3.51 ± 0.18	trace ^a^
Vanilic acid	26.1 ± 1.02	Trace ^a^	43.9 ± 1.36	44.1 ± 2.21	trace ^a^	trace ^a^
Caffeic acid	12.78 ± 0.59	1.57 ± 0.08	34.74 ± 0.52	9.40 ± 0.12	trace ^a^	trace ^a^
Syringic acid	98.60 ± 0.69	24.90 ± 0.05	121.54 ± 1.58	137.99 ± 1.24	49.35 ± 0.1	25.77 ± 0.05
*p*-Coumaric acid	1.19 ± 0.03	trace ^a^	trace ^a^	trace ^a^	trace ^a^	7.07 ± 0.05
Ferulic acid	4.65 ± 0.005	13.64 ± 0.67	6.94 ± 0.28	7.69 ± 0.08	6.66 ± 0.07	11.81 ± 0.01
Salicylic acid	4.32 ± 0.17	1.22 ± 0.01	5.18 ± 0.02	7.98 ± 0.07	0.12 ± 0.005	2.06 ± 0.03
Veratric acid	- ^b^	- ^b^	- ^b^	- ^b^	- ^b^	- ^b^
Synapic acid	78.50 ± 2.36	71.82 ± 0.43	107.08 ± 1.07	112.17 ± 1.79	84.80 ± 1.36	8.60 ± 0.09
3-OH-cinnamic acid	- ^b^	- ^b^	- ^b^	- ^b^	- ^b^	- ^b^
Rosmarinic acid	trace ^a^	trace ^a^	trace ^a^	trace ^a^	trace ^a^	trace ^a^
TOTAL	341.32	159.23	351.13	354.45	144.44	55.31

^a^ trace–trace amounts; - ^b^ not detected.

**Table 4:** Antioxidant activity–reducing power (RED), metal chelating power (CHEL), Inhibition of linoleic acid peroxidation (LPO) and free radicals scavenging ability (ABTS^•^) in methanol extracts from mistletoe berries harvested from various host trees.

Species	Host Trees	Antioxidant Activity (EC_50_ in mg mL^−1^ of extract)			
		RED	ABTS^•^	CHEL	LPO
*V. album*	*Fraxinus* *pensylvanica* Marsh.	1.04 ± 0.02 ^ns^	8.06 ± 0.12 ^ns^	0.88 ± 0.18 *	5.30 ± 0.03 *
	*Malus* *domestica* Borkh.	1.21 ± 0.02 ^ns^	13.03 ± 0.06 **	0.54 ± 0.03 ^ns^	7.55 ± 0.19 **
	*Sorbus* *aucuparia* L.	1.05 ± 0.01 ^ns^	7.72 ± 0.13 **	0.59 ± 0.10 ^ns^	6.60 ± 0.33 *
	*Populus* *nigra* ‘Italica’ L.	1.08 ± 0.01	8.17 ± 0.02	0.54 ± 0.03	5.65 ± 0.44
	*Tilia cordata* Mill.	0.92 ± 0.03 ^ns^	11.31 ± 0.14 **	0.45 ± 0.10 ^ns^	5.57 ± 0.43 ^ns^
*V. austriacum*	*Pinus sylvestris* L.	1.33 ± 0.02 ^ns^	14.77 ± 0.25 **	0.60 ± 0.01 ^ns^	10.24 ± 0.25 **

Values are presented in mean ± SEM, *n* = 3 and evaluated by one-way ANOVA (post test: Tukey) to compare each extract to *Populus nigra* ‘Italica’ L. extract. Differences were considered to be statistically significant if *p* < 0.05; ns—no significant differences; * *p* < 0.05; ** *p* < 0.01.

**Table 5:** Pearson correlation coefficient and R^2^ coefficient showing relationship between total phenolic content (TPC), total flavonoid content (TFC) total phenolic acids content (LC-ESI-MS/MS), total flavonoid aglycones (LC-ESI-MS/MS) and EC_50_ (ABTS^•^) for mistletoes berries extracts from different host trees.

Content/EC_50_	R (X,Y)	R^2^
TPC & EC_50_ (ABTS^•^)	−0.70	0.49
TFC & EC_50_ (ABTS^•^)	−0.81	0.65
Total phenolic acids content (LC-ESI-MS/MS) & EC_50_ (ABTS^•^)	−0.97	0.95
Total flavonoid aglycones content (LC-ESI-MS/MS) & EC_50_ (ABTS^•^)	−0.07	0.01

**Table 6:** Summary of optimized parameters for the quantitative analysis of flavonoid aglycones.

Compound	Retention Time (min)	Q1 (*m*/*z*)	Q3 (*m*/*z*)	DP ^a^ (V)	EP ^b^ (V)	CEP ^c^ (V)	CE ^d^ (eV)	CXP ^e^ (V)
Taxifolin	6.17	302.7	124.9	−45	−3.5	−18	−26	0
			284.8	−45	−3.5	−18	−14	−4
Myricetin	6.47	316.7	136.9	−55	−9	−14	−32	0
			150.9	−55	−9	−14	−26	0
Morin	6.82	300.7	124.9	−50	−3.5	−20	−24	0
			106.9	−50	−3.5	−20	−30	0
Eriodictiol	7.07	286.7	134.9	−45	−6	−12	−32	0
			150.9	−45	−6	−12	−18	−2
Luteolin	7.08	284.7	132.9	−75	−9	−18	−38	0
			150.9	−75	−9	−18	−26	0
Quercetin	7.13	300.7	150.9	−60	−2.5	−12	−26	0
			178.8	−60	−2.5	−12	−20	−2
3-*O*-Methylquercetin	7.32	314.7	299.8	−55	−9.5	−22	−18	−4
			270.8	−55	−9.5	−22	−26	−4
Apigenin	7.77	268.8	117	−70	−9.5	−12	−44	0
			106.8	−70	−9.5	−12	−34	0
Naringenin	7.81	270.8	119	−50	−11.5	−12	−34	0
			150.9	−50	−11.5	−12	−22	0
Kaempferol	7.94	284.7	116.8	−70	−5	−12	−46	0
			93	−70	−5	−12	−52	0
Isorhamnetin	8.09	314.7	299.7	−65	−2.5	−26	−20	−4
			150.9	−65	−2.5	−26	−30	0
Rhamnetin	8.85	314.7	165	−60	−5.5	−18	−24	0
			120.9	−60	−5.5	−18	−36	0
Chrysin	9.83	252.8	208.9	−80	−10	−14	−22	−2
			142.9	−80	−10	−14	−26	0
Sakuranetin	9.89	284.7	118.9	−60	−5.5	−12	−34	0
			164.8	-60	-5.5	−12	−20	−2
Prunetin	10.18	282.8	267.7	−55	−12	−18	−20	−4
			238.7	−55	−12	−18	−26	−2
Rhamnazin	10.31	328.7	270.8	−70	−3	−28	−26	−2
			313.8	−70	−3	−28	−14	−4

^a^ DP–Declustering Potential; - ^b^ EP–Entrance Potential; ^c^ CEP–Cell Entrance Potential; ^d^ CE–Collision Energy; ^e^ CXP–Collision Cell Exit Potential.

**Table 7:** Analytical parameters of LC-MS/MS quantitative method.

Compound	LOD (ng/µL)	LOQ (ng/µL)	R^2^	Linearity Range (ng/µL)
Taxifolin	0.015	0.05	0.9972	0.05–5
Myricetin	0.002	0.004	0.9959	0.011–3.6
Morin	0.0015	0.003	0.9951	0.01–5
Eriodictiol	0.005	0.015	0.9976	0.015–5
Luteolin	0.024	0.04	0.9961	0.04–4
Quercetin	0.0015	0.003	0.9960	0.031–3.1
3-*O*-Methylquercetin	0.001	0.002	0.9962	0.011–3.7
Apigenin	0.003	0.004	0.9952	0.012–6.3
Naringenin	0.025	0.033	0.9959	0.033–3.3
Kaempferol	0.02	0.033	0.9973	0.033–20
Isorhamnetin	0.012	0.024	0.9965	0.04–60
Rhamnetin	0.002	0.006	0.9955	0.006–0.625
Chrysin	0.025	0.042	0.9954	0.042–2.5
Sakuranetin	0.034	0.046	0.9955	0.072–7.2
Prunetin	0.05	0.075	0.9967	0.2–20
Rhamnazin	0.054	0.072	0.9962	0.072–7.2
